# Repeated Isoflurane Exposures Impair Long-Term Potentiation and Increase Basal GABAergic Activity in the Basolateral Amygdala

**DOI:** 10.1155/2016/8524560

**Published:** 2016-05-23

**Authors:** Robert P. Long II, Vassiliki Aroniadou-Anderjaska, Eric M. Prager, Volodymyr I. Pidoplichko, Taiza H. Figueiredo, Maria F. M. Braga

**Affiliations:** ^1^Department of Anatomy, Physiology, and Genetics, F. Edward Hébert School of Medicine, Uniformed Services University of the Health Sciences, 4301 Jones Bridge Road, Bethesda, MD 20814, USA; ^2^Program in Neuroscience, F. Edward Hébert School of Medicine, Uniformed Services University of the Health Sciences, 4301 Jones Bridge Road, Bethesda, MD 20814, USA; ^3^Department of Psychiatry, F. Edward Hébert School of Medicine, Uniformed Services University of the Health Sciences, 4301 Jones Bridge Road, Bethesda, MD 20814, USA

## Abstract

After surgery requiring general anesthesia, patients often experience emotional disturbances, but it is unclear if this is due to anesthetic exposure. In the present study, we examined whether isoflurane anesthesia produces long-term pathophysiological alterations in the basolateral amygdala (BLA), a brain region that plays a central role in emotional behavior. Ten-week-old, male rats were administered either a single, 1 h long isoflurane (1.5%) anesthesia or three, 1 h long isoflurane exposures, separated by 48 h. Long-term potentiation (LTP) and spontaneous GABAergic activity in the BLA were studied 1 day, 1 week, and 1 month later. Single isoflurane anesthesia had no significant effect on the magnitude of LTP. In contrast, after repeated isoflurane exposures, LTP was dramatically impaired at both 1 day and 1 week after the last exposure but was restored by 1 month after the exposures. Spontaneous GABA_A_ receptor-mediated IPSCs were increased at 1 day and 1 week after repeated exposures but had returned to control levels by 1 month after exposure. Thus, repeated exposures to isoflurane cause a long-lasting—but not permanent—impairment of synaptic plasticity in the BLA, which could be due to increased basal GABAergic activity. These pathophysiological alterations may produce emotional disturbances and impaired fear-related learning.

## 1. Introduction

Behavioral deficits, particularly of cognitive nature such as impairments in learning and memory, are common in patients following surgery under general anesthesia and have been collectively termed “postoperative cognitive dysfunction” [1, 2]. For the most part, these deficits are not permanent [1, 2] but may last long enough to significantly affect the ability to return to work or duty, as well as the overall quality of life. The causes of postoperative cognitive dysfunction are unclear [3], but animal studies indicate that general anesthesia alone can produce lasting cognitive deficits [4–7], which correlate with pathological and pathophysiological alterations in the hippocampus [5, 7, 8]. It has been reported that general anesthesia alone (without surgery) also produces psychological disturbances and negative mood effects, in young men; increased anxiety lasted for at least 4 days after anesthetic exposure, and depressive symptoms were still present at 30 days after exposure [9]. Postoperative anxiety and depression appear to be common [10, 11], and one can find a plethora of articles and discussions on the internet related to this issue, with speculations on the role of anesthesia. However, there is a scarcity of scientific data that can shed light on the extent to which postoperative emotional disturbances can be attributed to factors such as pain and discomfort, dissatisfaction with the outcome of the surgery, and/or worry about long-term outcome and implications on quality of life, or if general anesthesia can indeed induce long-lasting alterations in brain regions that regulate emotional behavior, thus causing or contributing to postoperative emotional dysfunction.

The amygdala is a brain region that plays a key role in emotional behavior [12, 13]. Experimental evidence suggests amygdala dysfunction after exposure to anesthetics. Thus, fear conditioning, which is primarily mediated by the amygdala [14–17], is impaired after general anesthesia, in rats [5] and mice [6]. The cellular mechanisms underlying fear conditioning and fear-related learning involve synaptic plasticity in the amygdala—and in particular in the basolateral nucleus of the amygdala (BLA)—in the form of long-term potentiation (LTP) of synaptic transmission [17–21]. There have been no studies so far examining if anesthetic exposure affects the synaptic capacity for LTP in the amygdala. In the present study, we investigated whether single or repeated exposures to isoflurane, a commonly used anesthetic in the clinical setting and in experimental animals, produce long-lasting impairments in LTP in the BLA. We also examined if basal GABAergic activity is altered, as GABAergic inhibition plays a significant role in the modulation of synaptic plasticity and LTP [18, 19, 22, 23], as well as in the excitability and function of the amygdala [24–28].

## 2. Materials and Methods

### 2.1. Animals

All animal experiments were conducted following the Guide for the Care and Use of Laboratory Animals (Institute of Laboratory Animal Resources, National Research Council) and were approved by the Uniformed Services University of the Health Sciences Institutional Animal Care and Use Committee. Experiments were performed using 10-week-old (~375 g) male, Sprague Dawley rats (Taconic Farms, Derwood, MD). Every effort was made to minimize animal suffering and reduce the number of animals used. Animals were pair housed in an environmentally controlled room (20–23°C, ~44% humidity, 12 h light/12 h dark cycle [350–400 lux], lights on at 6:00 am), with food (Harlan Teklad Global Diet 2018, 18% protein rodent diet; Harlan Laboratories; Indianapolis, IN) and water available* ad libitum*. Animals were given 5 days to habituate to the new environment after delivery.

### 2.2. Anesthesia

Anesthesia was induced using 3–5% isoflurane (Baxter, Deerfield, IL), at a fresh gas flow of 5–7 L/min, in a clear acrylic rodent anesthesia chamber (9′′ × 9.5′′ × 17.75′′, Kent Scientific, Torrington, CT). Once animals lost the ability to maintain an upright posture, the concentration of isoflurane was decreased to 3% for 3 min. Upon the absence of foot pinch, tail pinch reflex, or purposeful movement, anesthesia was reduced and maintained at 1.5 ± .5% for one hour. Minimum alveolar concentration was monitored by observing if the animals had a positive response to noxious stimuli. If there was a positive response, the anesthetic depth was increased by 0.1% and allowed to equilibrate for a period of 3~5 minutes. Animals were placed in a “neutral” spine orientation, and viscous saline solution (Akorn Inc., Lake Forest, IL) was applied to the animals' eyes bilaterally. Heart rate and blood oxygen saturation (pulse oximetry) were recorded using the SurgiVet V90043 (Smith Medical, Dublin, OH), while end-tidal oxygen and agent were monitored, throughout the anesthetic exposure, using the Agilent M1026A airway gas analyzer (Philips Healthcare, Andover, MA). Isoflurane was discontinued after 60 min. Once animals were able to maintain upright posture with purposeful movements, they were returned to their home cage. Animals in the experimental groups received either one isoflurane exposure or three isoflurane exposures, with a period of 48 hours between exposures, following the protocol described above.

### 2.3. Electrophysiological Experiments

Animals who received either single isoflurane exposure or repeated exposures were anesthetized with 3–5% isoflurane and rapidly decapitated, 1 day (about 24 h) or 1 week later. Rats who received repeated isoflurane exposures were also studied 1 month after the last anesthesia. Brain slices containing the amygdala were prepared as described previously [29]. Briefly, coronal slices (400 *μ*m) were cut using a vibratome (Leica VT 1200 S; Leica Microsystems, Buffalo Grove, IL), in ice-cold cutting solution consisting of (in mM) 115 sucrose, 70 NMDG, 1 KCl, 2 CaCl_2_, 4 MgCl_2_, 1.25 NaH_2_PO_4_, and 30 NaHCO_3_. Slices were transferred to a holding chamber, at room temperature, in a bath solution (artificial cerebrospinal fluid; ACSF) containing (in mM) 125 NaCl, 3 KCl, 1.25 NaH_2_PO_4_, 21 NaHCO_3_, 2 CaCl_2_ 1.5 MgCl_2_, and 11 D-glucose (all purchased from Sigma-Aldrich, St. Louis, MO). Recording solution was the same as the ACSF. All solutions were saturated with 95% O_2_ and 5% CO_2_ to achieve a pH near 7.4.

Field potential recordings were obtained in an interface-type chamber, maintained at 32~33°C, with a flow rate of the ACSF at ~1.5 mL/min, as described previously [30]. Field potentials were evoked in the BLA by stimulation of the external capsule, at 0.05 Hz, using a bipolar concentric stimulating electrode made of tungsten (World Precision Instruments, Sarasota, FL). For LTP experiments, a 20 min baseline was recorded followed by high-frequency stimulation (HFS; 3 trains of pulses at 100 Hz, each train lasted 1 s, and the interval between trains was 20 sec). After delivery of HFS, field potential recordings resumed with stimulation at 0.05 Hz. Recording glass pipettes were filled with ACSF and had a resistance of approximately 5 MΩ. Signals were digitized using the pClamp 10.4 software (Molecular Devices, Union City, CA) and analyzed using AxoGraph (AxoGraph X, Berkley, CA), and final presentation was prepared using GraphPad Prism (GraphPad Software, La Jolla, CA).

For whole-cell recordings, the slice chamber (0.7 mL capacity) had continuously flowing ACSF (~8 mL/min), at temperature 31~32°C. Tight-seal (over 1 GΩ) whole-cell recordings were obtained from principal neurons in the BLA. The neurons were visualized with an infrared light, through a 40x water immersion objective with a CCD-100 camera affixed (Dage-MTI, Michigan City, IN), using Nomarski optics in an upright microscope (Zeiss Axioscope 2, Thornwood, NY). The patch electrodes were filled with (in mM) 60 CsCH_3_SO_3_, 60 KCH_3_SO_3_, 5 KCl, 10 EGTA, 10 HEPES, 5 Mg-ATP, and 0.3 Na_3_GTP (290 mOsm; pH 7.2). Access resistance (15–24 MΩ) was regularly monitored during recordings, and cells were rejected if the resistance changed by more than 15% during the experiment. Spontaneous inhibitory postsynaptic currents (sIPSCs) were recorded in the presence of kynurenic acid, an NMDA and AMPA/kainate receptor antagonist, SCH50911, a GABA_B_ receptor antagonist, and LY341495, a metabotropic glutamate group II/III receptor antagonist (all from Tocris, Ellisville, MO), at a holding potential of +30 mV; these sIPSCs are mediated by GABA_A_ receptors, as they are blocked by the GABA_A_ receptor antagonist bicuculline methiodide [31]. Ionic currents were amplified and filtered (1 kHz) using the Axopatch 200B amplifier (Axon Instruments, Foster City, CA), digitally sampled (up to 2 kHz) using the pClamp 10.2 software (Molecular Devices, Sunnyvale, CA), and further analyzed using the Mini Analysis program (Synaptosoft Inc., Fort Lee, NJ) and Origin (OriginLab Corporation, Northampton, MA).

### 2.4. Statistics

Data are expressed as mean ± standard error (SEM). One way ANOVA with a Tukey post hoc test was used to compare the results from the LTP experiments. Whole-cell recording results were analyzed using Student's *t*-test. Statistical analyses were made using the software package PAWS SPSS 23 (IBM, Armonk, NY, USA). Differences were considered significant when *P* < 0.05. Sample size “*n*” refers to the number of slices (LTP experiments) or cells (whole-cell recordings); one to two slices were used from each rat in the LTP experiments, and 2 to 4 cells were used from each rat in the IPSC recordings.

## 3. Results

### 3.1. Effects of Single or Repeated Isoflurane Exposures on LTP in the BLA

Field potentials evoked by stimulation of the external capsule were recorded in the BLA, in slices obtained from control rats and rats who had been exposed to isoflurane. Stimulus intensity was adjusted to evoke a response of 65 to 75% of the maximum response amplitude. The magnitude of LTP was determined by averaging the amplitude of the evoked responses at 50 to 60 min after HFS and expressing it as a percentage of the baseline amplitude (before HFS).

**Figure 1:**
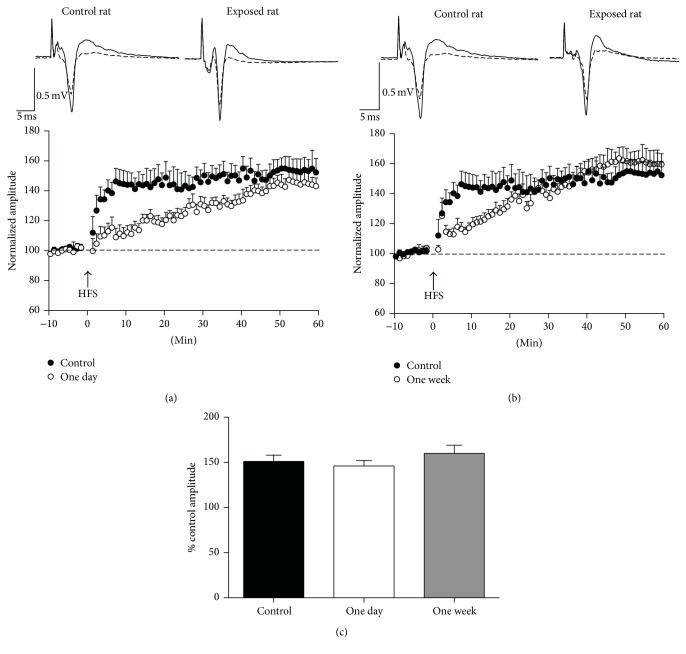
Single isoflurane exposure for 1 h had no significant effect on the magnitude of LTP in the BLA. Field potentials were evoked in the BLA by stimulation of the external capsule. (a) and (b) show the time course of the changes in the amplitude of the responses after high-frequency stimulation (HFS), in control rats (black circles) and rats exposed to a single, 1 h long isoflurane anesthesia (open circles), 1 day (a), and 1 week (b) after the exposure. The amplitudes of three responses recorded in each min (stimulation at 0.05 Hz) were averaged, and each data point on the plot is the mean and standard error of these averages, from 9 to 11 slices. Traces over the plots are examples from a control rat and from isoflurane-exposed rats; the superimposed field potentials are a baseline response and a response at 50 to 60 min after HFS (each trace is the average of 10 to 20 sweeps). (c) Group data of the mean amplitudes during the 50 to 60 min time window after HFS, expressed as % of the control (baseline) amplitude. Sample sizes, *n* = 10 for the control group, *n* = 9 for the 1-day group, and *n* = 11 for the 1-week group.

After single exposure to isoflurane, LTP had a slower time course compared to the controls, but its magnitude did not differ from the controls, 50 to 60 min after HFS (Figure 1). Thus, compared to the percent change in the response amplitude after HFS in the control animals (151 ± 7% of the baseline amplitude, from 0.44 ± 0.04 mV at baseline to 0.66 ± 0.06 mV after HFS; *n* = 10), the percent change in the response amplitude of the rats who received a single isoflurane exposure was not significantly different (*F*_(2,29)_ = 1.01, *P* = 0.377) at 1 day (146 ± 6%, from 0.55 ± 0.09 mV at baseline to 0.80 ± 0.13 mV after HFS; *n* = 9; *P* = 0.840; Figures 1(a) and 1(c)) or 1 week (160 ± 9%, from 0.47 ± 0.01 at baseline to 0.76 ± 0.03 mV after HFS; *n* = 11; *P* = 0.675; Figures 1(b) and 1(c)) after the exposure.

**Figure 2:**
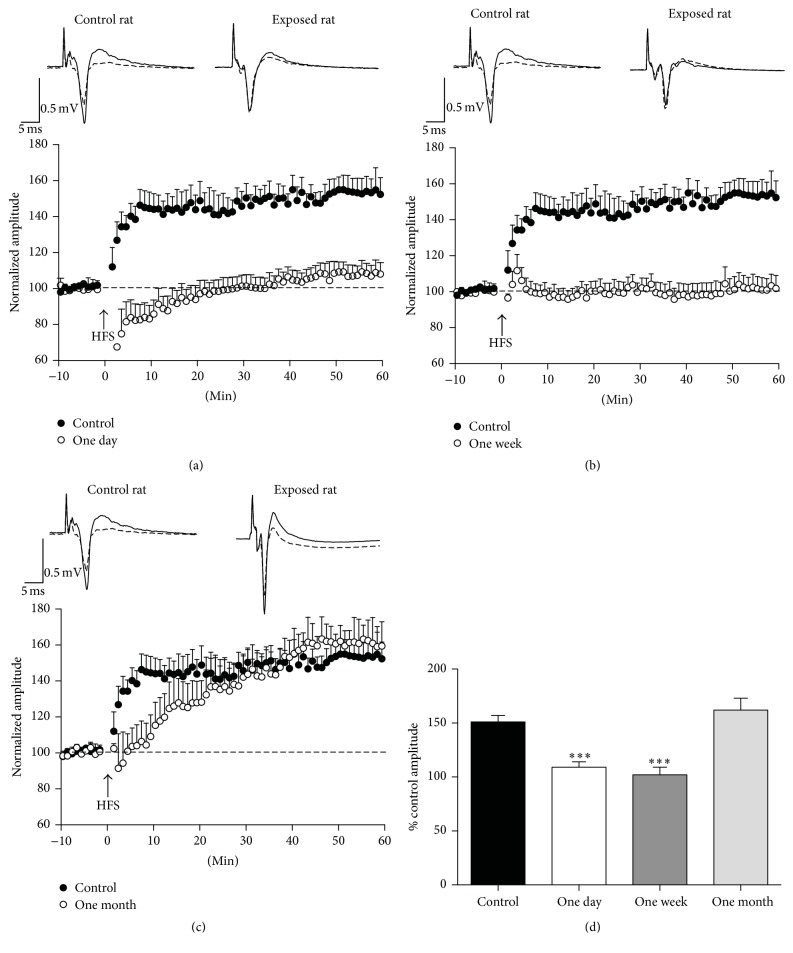
Effects of repeated isoflurane exposures on LTP in the BLA. Field potentials were evoked in the BLA by stimulation of the external capsule. (a), (b), and (c) show the time course of changes in the amplitude of the responses after high-frequency stimulation (HFS), in control rats (black circles) and rats exposed to three sessions of 1 h isoflurane anesthesia, with a 48 h interval between sessions (open circles); LTP studies were performed at 1 day (a), 1 week (b), and 1 month (c) after the last exposure. The amplitudes of three responses recorded in each min (stimulation at 0.05 Hz) were averaged, and each data point on the plot is the mean and standard error of these averages, from 9 to 13 slices. Traces over the plots are examples from a control rat and from isoflurane-exposed rats; the superimposed field potentials are a baseline response and a response at 50 to 60 min after HFS (each trace is the average of 10 to 20 sweeps). The data are summarized in (d), for the amplitude of the field potentials during the 50 to 60 min time-window after HFS, expressed as % of the control amplitude. ^*∗∗∗*^ *P* < 0.001, *n* = 10 for the controls, *n* = 10 for the 1-day group, *n* = 13 for the 1-week group, and *n* = 9 for the 1-month group.

After repeated exposures to isoflurane, LTP was significantly inhibited at both 1 day and 1 week after the last of the three exposures (Figure 2). Thus, compared to the percent change in the response amplitude after HFS in the control animals (151 ± 7%, from 0.44 ± 0.04 mV at baseline to 0.66 ± 0.06 mV after HFS; *n* = 10), the percent change in the response amplitude of the rats who received repeated isoflurane exposures was significantly smaller (*F*_(3,41)_ = 16.49, *P* < 0.001), at 1 day (109 ± 5%, from 0.51 ± 0.03 mV at baseline to 0.56 ± 0.04 mV; *n* = 10; *P* < 0.001; Figures 2(a) and 2(d)) and 1 week (102 ± 7%, from 0.46 ± 0.01 mV at baseline to 0.47 ± 0.04 mV after HFS; *n* = 13; *P* < 0.001; Figures 2(b) and 2(d)) after the exposures. One month after the last of the three exposures, the magnitude of LTP was not different from that in the controls (162 ± 11%, from 0.46 ± 0.04 mV at baseline to 0.74 ± 0.07 mV after HFS; *n* = 9; *P* = 0.769; Figures 2(c) and 2(d)).

### 3.2. Effects of Repeated Isoflurane Exposure on Basal GABAergic Activity in the BLA

GABAergic inhibition significantly modulates synaptic plasticity, as it can suppress the induction and expression of LTP [18, 19, 22, 23]. Therefore, we examined whether the impaired synaptic plasticity in the BLA after repeated exposures to isoflurane was associated with alterations in spontaneous inhibitory activity. Whole-cell recordings were obtained from BLA principal neurons, in the presence of 1.5 mM kynurenic acid, 10 mM SCH50911, and 3 mM LY341495. To quantify the level of basal GABA_A_ receptor-mediated synaptic activity, we measured the frequency and amplitude of sIPSCs, as well as the total charge transferred by sIPSCs across the membrane. The charge, in pico Coulombs, was calculated as the area delimited by the inhibitory current and the baseline; current areas were analyzed for a time window of 20 s.

**Figure 3:**
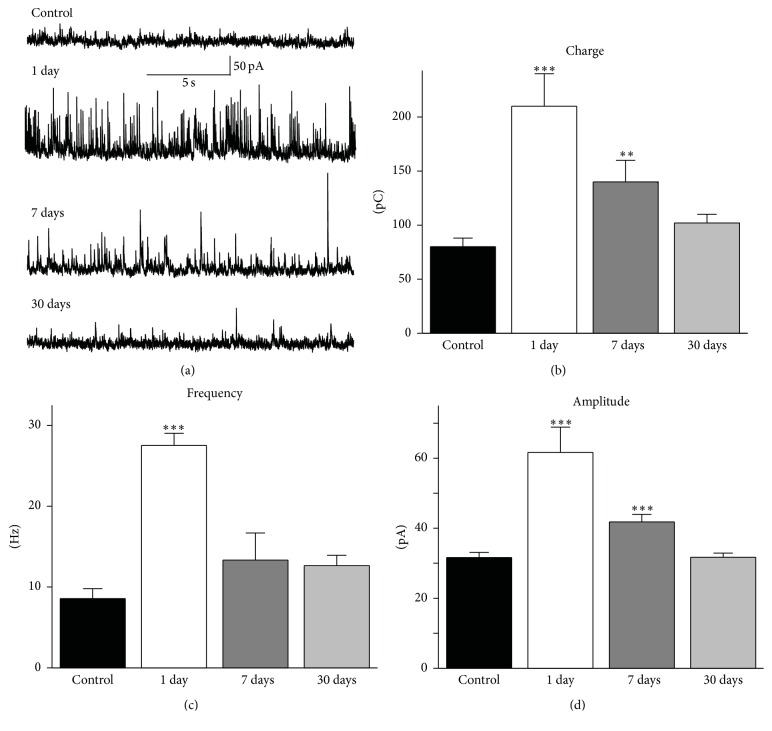
Effects of repeated isoflurane exposures on basal GABAergic synaptic activity in the BLA. Whole-cell recordings of spontaneous IPSCs (sIPSCs) were obtained from principal neurons, at a holding potential of +30 mV. (a) Representative traces of sIPSCs recorded from a control rat (top trace) and from rats who received repeated exposures to isoflurane; recordings were obtained at 1 day, 1 week, and 1 month after the last of the three exposures. (b) Group data showing that the total charge carried by the recorded currents was increased at 1 day and 1 week after the last of the three exposures but returned to levels that did not differ significantly from the control, 1 month after the exposures. (c) Group data of the frequency of sIPSCs, which was significantly increased at 1 day after repeated exposures. (d) Group data of the amplitude of sIPSCs, which was significantly higher than that in the control group, at 1 day and 7 days after repeated exposures. ^*∗∗∗*^ *P* < 0.001 and ^*∗∗*^ *P* < 0.01; *n* = 13 for the control group; *n* = 17 for the 1-day group, *n* = 9 for the 1-week group; and *n* = 16 for the 1-month group.

One day after the last of three isoflurane exposures, the charge transferred by sIPSCs in BLA principal neurons was 210 ± 30 pC (*n* = 17), which was significantly greater than that in control animals (80 ± 8 pC, *n* = 13; *P* = 0.0003; Figures 3(a) and 3(b)). One week after the repeated exposures, the charge transferred by sIPSCs was 140 ± 20 pC (*n* = 9), which was also significantly greater compared to the controls (*P* = 0.002). One month after the repeated exposures, the charge transfer (102 ± 8 pC, *n* = 16) had returned to control levels (*P* = 0.07; Figures 3(a) and 3(b)). The frequency of sIPSCs was also significantly increased from 8.6 ± 1.2 Hz (*n* = 13) in the control group to 27.5 ± 1.5 Hz (*n* = 17) at 1 day after the repeated exposures (*P* < 0.0001; Figure 3(c)). One week after the repeated exposures, the sIPSC frequency was 13.3 ± 2.4 Hz (*n* = 9), and at the 30-day time point it was 12.6 ± 1.3 Hz (*n* = 16, *P* > 0.05; Figure 3(c)). The amplitude of sIPSCs was significantly increased from 31.6 ± 1.5 pA (*n* = 13) in the control group to 61.6 ± 7.2 pA (*n* = 17) at 1 day after the repeated exposures and 41.8 ± 2.1 pA (*n* = 9) at 1 week after the repeated exposures (*P* < 0.001; Figure 3(d)); at the 30-day time point the amplitude of sIPSCs had returned to control levels (31.7 ± 1.2 pA, *n* = 16, *P* = 0.96).

## 4. Discussion

The present study showed that after repeated exposures to isoflurane, but not after a single, 1 h exposure, synaptic plasticity in the BLA is impaired. Thus, HFS did not induce LTP at 1 day and 1 week after the last of three isoflurane exposures. The impairment in synaptic plasticity was accompanied by an increase in spontaneous GABAergic activity. One month after the repeated exposures, both the synaptic capacity for LTP and the basal GABAergic activity had returned to control levels.

It is well known that isoflurane and other anesthetics allosterically modulate postsynaptic GABA_A_ receptors and enhance inhibition [32, 33]. Thus, in the presence of isoflurane, the GABA_A_ receptor-mediated charge transfer is enhanced, owing mainly to prolongation of the decay of the inhibitory current; this has been demonstrated in evoked IPSCs in the BLA [34], as well as in miniature IPSCs in CA1 hippocampal neurons [35]. However, these effects are fully reversible upon washout of isoflurane [34]. The only piece of evidence suggesting long-term effects of isoflurane on GABAergic inhibition comes from studies in the mouse hippocampus, where the expression of the *α*5 subunit-containing GABA_A_ receptors and the tonic inhibitory currents mediated by these receptors were increased in CA1 pyramidal cells, for at least 1 week after* in vivo* exposure to isoflurane or etomidate [36]. The present study demonstrates that, in the rat BLA, repeated exposures to isoflurane produce a long-term increase of spontaneous GABA_A_ receptor-mediated synaptic transmission. It remains to be determined whether this increase is due to enhanced expression or sensitivity of postsynaptic GABA_A_ receptors on principal BLA neurons, or if it involves presynaptic effects on GABA release, or a long-lasting increase in the excitability of BLA interneurons.

The failure of HFS to induce LTP at 1 day and 1 week after repeated exposures could be due, at least in part, to the enhanced basal GABAergic inhibitory activity. The regulatory role of GABAergic inhibition over LTP of excitatory synaptic transmission [18, 19, 22, 23] has become evident since the early studies of LTP in* in vitro* brain slices, where it was often necessary to partially block GABA_A_ receptors in order to facilitate LTP induction; reduction of GABA_A_ receptor-mediated activity facilitates postsynaptic depolarization during HFS and Ca^++^ influx—via NMDA receptors in most synapses, including the amygdala [37, 38]—which is necessary for induction of LTP [39]. The increased inhibitory activity in the BLA network may have prevented sufficient postsynaptic depolarization and Ca^++^ influx for LTP induction. The pronounced, but transient, posttetanic depression one day after repeated exposures may suggest enhanced evoked inhibition, although presynaptic mechanisms may also be involved.

The results show that a single exposure to isoflurane was not sufficient to produce an inhibiting effect on LTP induction, since the increase in the amplitude of the evoked responses at 50 to 60 min after HFS was not statistically different from that of the control group. It is worth noting, however, that the time course of LTP expression was very slow, particularly at 1 day after single exposure, suggesting that isoflurane did have some effect. The early phase of LTP expression involves phosphorylation of AMPA receptors and trafficking of these receptors to postsynaptic sites [40, 41], while presynaptic mechanisms may also be involved [42]. Which of these mechanisms might have been affected by a single isoflurane exposure remains to be determined, but the effect appeared to be transient and did not prevent the long-term increase of the responses.

The BLA is a key part of the brain circuit involved in fear-related learning and fear conditioning [14–17]. At the cellular level, fear conditioning is associated with induction of LTP in the BLA [17–21]. Therefore, the impaired LTP in the BLA after repeated isoflurane exposures, as observed in the present study, suggests impairment in fear-related learning. Impaired fear conditioning after isoflurane exposure has been observed in adult mice [6] and rats [5], lasting at least for 2 weeks after exposure in the rat study [5]. Longer-lasting impairments in fear conditioning, persisting into adulthood, have been observed after exposure of neonatal mice to sevoflurane [43]. The present findings suggest that, in addition to pathophysiological alterations in the hippocampus [5], impaired synaptic plasticity in the BLA underlies the observed deficits in fear conditioning.

In addition to impairing synaptic plasticity and fear-related learning, the enhanced basal GABAergic inhibition in the BLA may also have other functional consequences. The amygdala plays a central role in assigning emotional value and processing the emotional significance of sensory information as well as in orchestrating the emotional component of the behavioral response [44, 45]. These processes may be affected by an enhanced basal GABAergic activity in the BLA, which would suppress the excitability of the network and the flow of information to the central amygdala and extra-amygdala targets. However, increased GABAergic activity in the BLA is not compatible with increased anxiety or depression, as these emotional derangements are associated with reduced GABAergic inhibition [26, 29, 30, 46, 47] and hyperexcitability of the amygdala [48–52]. There is little information on the nature of the emotional deficits after exposure to anesthetics. Adult mice did not show increased anxiety-like behavior in the open field [8] or the elevated plus maze [6], after 1 h isoflurane exposure. Prolonged anesthetic exposures in 6-day-old mice [43] or infant rhesus monkeys [53] produced low motivation and deficits in social interaction that persisted into adulthood. Human studies also have noted increased incidence of behavioral disorders if anesthesia/surgery takes place in early childhood [54], while symptoms indicative of depression have been reported in healthy young men, lasting at least 30 days after exposure to halothane or isoflurane for 6 to 7 h [9]. More studies are needed to provide a better understanding of the long-lasting effects of anesthetic exposure on emotional behavior, their age dependency, and the underlying mechanisms.

## 5. Conclusions

Repeated exposures to isoflurane can alter the physiology of the BLA, producing long-lasting increases in spontaneous GABAergic synaptic transmission and impairment in synaptic plasticity. Behaviorally, such alterations may translate into deficits in processing the emotional components of sensory experiences, as well as in acquiring and consolidating emotionally significant memory. Despite the long-lasting nature of the effects of repeated isoflurane anesthesia on the BLA, the results suggest that these effects are not permanent.
